# The family myth in nursing care for children in psychological distress*

**DOI:** 10.1590/1980-220X-REEUSP-2023-0414en

**Published:** 2024-05-20

**Authors:** Giulia Delfini, Vanessa Pellegrino Toledo, Ana Paula Rigon Francischetti Garcia

**Affiliations:** 1 Universidade Estadual de Campinas, Faculdade de Enfermagem, Campinas, SP, Brazil.

**Keywords:** Nursing Care, Child, Mental Health, Family, Psychoanalysis, Cuidados de Enfermería, Niño, Salud Mental, Familia, Psicoanálisis, Cuidados de Enfermagem, Criança, Saúde Mental, Família, Psicanálise

## Abstract

This is a theoretical study aimed at reflecting on the contribution of the concept of family myth to nursing care for children in psychological distress. It is methodologically structured around three topics: the importance of the family in caring for children; the perspective of family-centered nursing care for children in psychological distress; and the contribution of the understanding of family myth to nursing care for children in psychological distress. The following dialectic is considered: the family, considered by current literature to be a harmonious unit, also triggers family conflicts that can be the cause of psychological suffering. The concept of family myth emerges as a possible theoretical anchor for nursing care for children in psychological distress, as it allows nurses to consider the signifiers that mark the child’s psychological structure and construct their symptoms. Uncovering the place that the family assigns to the child enables nurses to help them construct and elaborate their own place as a subject in their subjectivity.

## RESUMO

Trata-se de um estudo teórico com o objetivo de refletir sobre a contribuição do conceito de mito familiar para o cuidado de enfermagem à criança em sofrimento psíquico, sendo metodologicamente estruturado em três tópicos: importância da família no cuidado de crianças; perspectiva do cuidado de enfermagem centrado na família de crianças em sofrimento psíquico; e contribuição do entendimento de mito familiar para o cuidado de enfermagem à criança em sofrimento psíquico. Considera-se a dialética: a família, tida pela literatura atual como unidade harmônica, também desencadeia conflitos familiares que podem ser a causa de sofrimentos psíquicos. O conceito de mito familiar surge como possibilidade de ancoragem teórica ao cuidado de enfermagem à criança em sofrimento psíquico ao permitir que o enfermeiro considere os significantes que marcam a estruturação psíquica da criança e constroem seu sintoma. Desvendar o lugar que a família destina à criança viabiliza ao enfermeiro auxiliá-la a construir e elaborar um local próprio enquanto sujeito em sua subjetividade.

## RESUMEN

Se trata de un estudio teórico dirigido a reflexionar sobre la contribución del concepto de mito familiar a los cuidados de enfermería a niños con sufrimiento psicológico. Metodológicamente se estructura en torno a tres temas: la importancia de la familia en el cuidado de los niños; la perspectiva de los cuidados de enfermería centrados en la familia para niños con sufrimiento psicológico; y la contribución de la comprensión del mito familiar a los cuidados de enfermería a niños con sufrimiento psicológico. Se plantea la dialéctica: la familia, considerada por la literatura actual como una unidad armoniosa, también desencadena conflictos familiares que pueden ser causa de sufrimiento psicológico. El concepto de mito familiar emerge como anclaje teórico para los cuidados de enfermería a los niños en malestar psicológico, ya que permite a las enfermeras considerar los significantes que marcan la estructura psicológica del niño y construyen sus síntomas. Desvelar el lugar que la familia asigna al niño permite a las enfermeras ayudarle a construir y elaborar su propio lugar como sujeto en su subjetividad.

## INTRODUCTION

The prevalence of mental disorders in children and adolescents is globally estimated at 13,4%, demonstrating that child and adolescent mental health is a worldwide public health problem, aggravated by the difficulty in accessing community services aimed at this type of care^(1,2)^.

In Brazil, the estimated prevalence of psychiatric disorders in the population of children and adolescents is 13,1%, which is close to the data found internationally^(3)^. In this context, the Child and Adolescent Psychosocial Care Centers (CAPSij in the Portuguese acronym) were created in 2002, as strategic services aimed at providing mental health care to children and adolescents in severe psychological distress, following the precepts of the psychosocial care model^(4)^.

The National Policy for Comprehensive Child Health Care (PNAISC in the Portuguese acronym) considered essential to have an environment that is favorable to the full growth and development of the subject’s physical, mental and emotional capacities and abilities, advocating that children be linked to their family context^(5)^. As a result, it is important that health teams listen to and look at both the child and the family, and the bond established between them^(5)^.

After the Psychiatric Reform, nurses, an essential piece of the multi-professional team, started to act as therapeutic agents during the nurse-patient relationship to build the nursing process, developing care by considering the subjectivity of the subject, understanding their experiences and helping them to reintegrate into social life^(6)^. In order to do this, the child must be seen as a subject who lives in a social environment made up of other individuals, especially their family members^(5)^.

Aiming in this direction, it is essential for nurses to base their care on a theoretical framework. One possibility that addresses the family in its relationship with the child is the psychoanalytic framework, which considers that psychic illnesses can originate in childhood, a period in which the psyche and ways of responding to the impasses that arise throughout life are structured^(7)^. Thus, even if psychological suffering is not present in childhood, it can emerge at any age from what is psychically structured in this phase.

In childhood children are constituted as speaking subjects through the insertion of fundamental signifiers that allow them to enter language^(7)^. This treasure trove of signifiers presented to the baby is called the family myth, a concept used in psychoanalysis to represent what the child breathes where it is placed, in other words, it is made up of valuable components for the family and society into which it will be introduced^(7,8)^.

Thus, nurses working in children’s mental health, who use psychoanalysis as a theoretical reference to establish care and the therapeutic relationship, act through transference with the aim of grasping the logic of the unconscious, taking into account the family myth in which the child is inserted^(6–8)^.

This study is justified by the high rates of mental disorders in the infant population and the difficulty in accessing community services aimed at this public, highlighting the need to consolidate comprehensive care for children, which can be carried out through nurse care for children in psychological distress, highlighting the importance of the family in child development and in the process of becoming ill^(1,2,5,7,8)^. Having said that, this study aims to reflect on the contribution of the concept of family myth to nursing care for children in psychological distress.

From a methodological point of view, this theoretical study is structured in three topics: the first aims to understand the importance of the family in caring for children; the second aims to describe the perspective of family-centered nursing care for children in the context of mental health; and finally, the third topic aims to reflect on how understanding the concept of family myth can contribute to nursing care for children in psychological distress.

## THE IMPORTANCE OF THE FAMILY IN CARING FOR CHILDREN

Taking into account the Brazilian context of child health since the late 1980s, children have benefited from policies and programs that seek to expand access to health services and ensure comprehensive care, encompassing their family and social context^(9)^.

The Statute of the Child and Adolescent, published in 1990, states that children have the right to be raised and educated in a family, in an environment that guarantees their full development^(10)^. The organization of actions and services for children and their families must be articulated with the health care network, leveraging available resources and ensuring continuity of comprehensive care^(5,9,11,12)^.

The PNAISC considers the family to be a fundamental axis to be included in care, since it considers early childhood, between the ages of zero and five, to be decisive for the healthy development of human beings, while family experiences can have influences throughout life, both positive and negative^(5)^.

It argues that the basis of the subject’s mental health is built during the period of psychological development, beginning at birth and extending until the point at which the child recognizes itself as a subject distinct from the mother, father and other members of the family^(5,12)^. In order to build this autonomy, the family must also be the target of care^(5,9,11,12)^.

It also considers it essential to strengthen family ties, recognizing that the relationship between mother and baby, the family’s care for the child and how it is received and addressed to the world, are fundamental factors for its healthy development^(5)^. Thus, intervention with children should involve family members, guiding them on the continuity of care in the home environment and offering the necessary support to the whole family^(5,12)^.

The Family Health Strategy (FHS) is the gateway to the health care networks of the Unified Health System (SUS) and one of the most important points of the Psychosocial Care Network (RAPS in the Portuguese acronym), as it advocates and enables comprehensive health care^(11–13)^. It is through this network that mental health care finds possibilities for welcoming, incorporating, structuring and developing it, and which enhances the construction of new spaces for producing knowledge and interventions^(12)^.

Thus, considering the network of care, the FHS and CAPSij are the main devices for child mental health care, with the child’s protagonism as a priority, being considered in their family dynamics^(13)^.

Recognizing the role of the family in the child’s care helps to maintain and promote the bond, building a relationship of trust between the user and the health service^(5,14)^. It is necessary to respect the values, beliefs, principles and fragilities of the family environment, with the aim of providing the best way of conducting care^(14)^.

Therefore, health professionals must understand the family as a whole, because what affects one of its members can affect the entire family system and, consequently, have a direct influence on the child’s health^(11,14)^. Thus, a bond must be established not only with the children, but also with their family, with the aim of reducing the risk of the child becoming ill, strengthening the family and contributing to the promotion of the child’s development and treatment^(5,14)^.

The importance of including the family in the care of children in psychological distress is clear, as evidenced by the inclusion of family members in policies and services aimed at treating this population^(5,9,11–14)^.

## PERSPECTIVE ON FAMILY-CENTERED NURSING CARE FOR CHILDREN IN PSYCHOLOGICAL DISTRESS

This topic will present a possible reading of family-centered nursing care for children in psychological distress, an approach currently considered in policies and literature. Nurses are central to providing comprehensive care for children, as they develop bonds and work together with them and their families, taking into account the environment in which they live, which enables comprehensive and systematized care^(11)^.

One of the main recommendations for nursing care in children’s mental health is to work in partnership with parents and/or family members, considering family-centered care by involving them in the treatment and developing a relationship of trust^(5,12,13)^. Some authors even point out the need to sometimes direct nursing care to family members^(13)^.

By working in partnership with parents/family members, the activities are in line with the principles of the biopsychosocial healthcare model, which advocates family-centered care developed with respect for human dignity, which can have a positive impact on psychosocial rehabilitation and enable autonomy, as well as training and empowering individuals^(15)^.

Pediatric nursing, as a modality of care, also argues that it is essential to include the family in child care, allowing them to be present and actively involved^(16)^. To this end, nurses must recognize the vital role of the family, aiming to plan and care for the child and family members in a mutually beneficial partnership, understanding them as unique beings, recognizing their singularity and totality, and ensuring stability in the nurse-child-family relationship^(16)^.

In this context, nurses must seek to understand families’ narratives and how treatment can best meet their needs, developing relationships with family members with the aim of creating health-promoting environments for the child^(2)^.

In order to make this possible, the buildup of care systems is intended with the aim of reducing disparities and facilitating access, and nurses are essential for strengthening and expanding this type of care, considering that the possibility of children entering health networks may be places where their families interact naturally, such as schools^(2)^.

Thus, for nursing care in children’s mental health to be centered on the family, it is necessary to consider the family as a unit of care, with the main objectives of stimulating their bond with the child and ensuring the participation of family members in the planning of health actions^(5,12,14,16)^. In other words, it consists of a communicative act between the professional, the child and their family^(16)^.

It is therefore extremely important for nurses to include the family in their practice, since their support and involvement increases the quality of care for children in psychological distress^(17)^. By including family members, professionals help them to make sense of their suffering, construct new meanings for the health-disease process, expand their knowledge and enhance their skills in the context of care^(17)^.

Current policies and literature on nursing care in children’s mental health focus on family-centered care, which is called upon to build therapeutic projects^(11–13)^. The family is therefore seen as an idealized unit in which harmony and balance reign, a possibility for consolidating partnerships with health services^(18)^.

However, the aim of this article is to develop nursing care for children in psychological distress in their own unique way, taking into account the family context in which they live. For Lacan, the family is seen as the place of the most stable and typical complexes, called family complexes, which are permeated with conflicts and promote the psychic disputes that make it possible for a singular subject to emerge^(18,19)^. In other words, when faced with the castration, frustration and deprivation that the family universe imposes on them, the child builds and uses their symbolic arsenal to signify their place in this family dynamic, which opens up the possibility of structuring the subject^(18,19)^.

Therefore, the children’s demands must be heard in their singularity, as it marks a particular expression of this being who occupies a place in the established family complex^(18)^. Family conflicts, in this context, are not seen as an obstacle to the structuring of the child, but rather as “organizers” of psychic development, necessary for the emergence of a subject and capable of producing health through their elaboration^(18,19)^.

While family complexes are described as psychic disputes, myth is the narrative of these disputes^(19)^. Armed with these two concepts, the psychoanalyst Ricardo Rodulfo formulated the concept of family myth, which was taken into account in this study and will be discussed at length in the following section.

## CONTRIBUTION OF THE UNDERSTANDING OF FAMILY MYTH TO NURSING CARE FOR CHILDREN IN PSYCHOLOGICAL DISTRESS

To define a child is, first of all, to circumscribe the signifiers that have a close relationship with them, which are produced by those around them^(8)^. In other words, in order to work in the clinic with children in psychological distress, it is necessary to look at their family history, since what precedes this little being is decisive for them, even before they exist^(8)^.

At birth, the baby requires a caregiver who will act as an interpreter of language in the role of the Other, also called the maternal function, which names the newborn’s actions as it interprets their state and produces meanings capable of soothing them^(7)^. It’s worth pointing out that the term “maternal function” doesn’t mean that this task is the mother’s alone, but rather that it’s carried out by all those who surround the baby and play the role of inserting them into language^(7)^. It is necessary to demystify the centrality of the family by understanding that there are other possible ties in the subjective organization of a child and to consider that the maternal role can be exercised by other caregivers, especially in the Brazilian context, where there is not always the presence of a father, but there are grandparents and aunts, for example.

The children will be inserted into the logic of language as they enter a social order that precedes them, represented by the signifiers offered to them^(7,8)^. In other words, the social dimension of the family is key to the constitution of the subject of the unconscious, because there is an established symbolic order and a system of pre-existing relationships of a signifying order that precedes it, since the Other that precedes it is already taken over by language^(7,8)^. As a result, the child makes use of the signifiers found in the Other, articulated in a chain, in order to find a place to inscribe themselves and name their experiences and sensations^(6)^.

For the abovementioned reasons, in order to work with children in psychological distress, nurses need to consider not only their first years of life, but their pre-history towards previous generations, i.e. where they haven’t been before, the history of the family^(8)^.

The family’s place in this context is to offer the baby signifiers, which will be unpredictably taken as their own^(8)^. In other words, the family myth does not have the function of decreeing a univocal direction for the child, which the child lacks because its paths are unpredictable, and the nurse must observe the child’s choice of direction without pretending to know which aspects of this myth it will take as its own and which it will reject^(8)^. The variation and abundance of signifiers offered to a child, even before birth, is closely related to the position they will occupy in the family dynamic^(8)^.

In this process, the Other, as a treasure trove of signifiers, enables the formation of a signifying structure made up of elements that are significant to the family and society in which the baby is inserted, which is equivalent to the concept of family myth^(7,8)^. This comprises real or phantasmatic knowledge that each new family member is confronted with, i.e. it is essential for understanding the child, who is marked by it^(20)^.

It is through the family myth that the signifiers are presented to the child in their first moment of self-elaboration, through daily practices that include acts, sayings, educational norms, body regulations, among others, and it is impossible to predict which elements will predominate in their psychic constitution^(8)^. This myth receives them and sculpts their nascent drives, giving access to life, civilization and desires, and is closely related to the arrangement of possible suffering, in other words, it can be the origin and cause of psychic problems^(20)^.

During clinical work with children in psychological distress, nurses need to understand that the family myth does not appear as a finished unit, as it is not a congruent, unitary, systematized and harmonious material, and is complex to visualize^(8)^. It is more like a network of small myths, and the professional has to deduce it over the course of the treatment, through more or less enlightening phrases and fragments, extracting pieces as they go through its incongruities, contradictions, gaps and dissociations^(8)^.

It’s worth pointing out that the family myth as a place where the child searches for signifiers is, first and foremost, the maternal body as a matrix housing the signifiers of openness, since corporeality is conceived from the Other of language^(8,21)^. The newborns are then taken by this maternal other who signifies their existence, in a dialectical movement in which they desire, taking them as objects of their own desire^(20)^. The term “maternal body” should be considered as the place where the treasure trove of signifiers is found, in other words, it can be attributed to various family members and is not exclusive to the mother^(8,21)^.

The maternal body is therefore presented to the child as the universal continent of all primordial objects in primitive disorder, filled with diverse, shattered and fragmented materials^(8,22)^. The experience of the child’s relationship with this body will teach them to assimilate the plurality presented to them, which will open the way to forming their own unity^(8,22)^. For this to happen, it is essential that the Other inserts the babies into language and inscribes them in an organic body, ensuring the structuring of their psychic apparatus^(7,8,22)^.

As a result, there is nothing in a subject’s body that is not inhabited by language, so that even in the womb the baby is inserted into it, as it participates in the maternal body, made up of its own signifiers^(21)^. The child, in the real of its body, is involved in the symbolic of the language that preexists it, appearing as a real event in a field that belongs to the Other^(8,21,22)^.

This body of the Other, as has already been said, is the depository of signifiers par excellence, and it will be in the exchanges between mother and child that these will be inscribed on the baby’s body^(8,21)^. The latter, in turn, offers its body to someone who takes care of it, cleans it and names it, enabling the emergence of a relationship in which the baby is equivalent to the phallus, which allows the birth of a desiring subject, the subject of the unconscious^(20)^.

The transmission of this family myth, derived from the maternal body, takes place through narratives, concrete interventions, caresses and intonations which, through repetition, become significant, through touch, hearing, proximity, warmth or distance of contact^(8)^. Thus, the maternal body is the family myth and, if the child is favorably disposed towards it, interacting satisfactorily in the universe of insignia represented by the behaviors of those who exercise the maternal function, they will be able to install their tendencies, drives and desires^(8,19,22)^.

From this encounter with the maternal body, permeated by the family myth that entrenches it and colors its attitudes, positions, sayings and fantasies, with the baby’s body, an imagined body will emerge, represented by a place prepared, in a symbolic world, for the child to live^(8)^.

As the mother establishes a bond with the baby, she situates it as a subject who, through a specular relationship, makes it possible for it to construct an imaginary self, giving contour and unifying what was shattered as a non-image of this body^(20)^. In this way, a relationship of corporeality is established, making it possible for a body to emerge from the real into an imaginary and symbolized body^(20)^.

As a result, the baby’s place of inscription is the family discourse, and the encounter between his/her body and his/her mother’s, through touch, voice and gaze, allows to become a subject of language, which enables him to express himself through words^(7,8,20,21)^. In other words, the baby takes from the body of the Other, by extracting the signifiers of the family myth that are essential for its constitution, the materials it needs to rise to the symbolic order of intersubjectivity^(8)^.

It is therefore of the utmost importance that the baby takes on the active task of burrowing into the discourse of the Other, a process that consists of finding and extracting signifiers that represent it in relation to and within the desiring field of family discourse, an action that is necessary to be constituted as a subject in a body of its own and not be the passive object of his/her prehistory^(8)^.

It is also essential that the children, from a certain age, perceive and look for contradictions in this family myth that surrounds them, so that they can produce ruptures that put consolidated versions to the test, in other words, they need to question^(8)^. In this way, it won’t respond passively to the ways in which those around it invest and will be constituted as a subject who actively addresses the impasses arising from the family myth that precedes it^(20)^.

Against this backdrop, nurses need to consider that the baby is invested, designated and desired by its parents and family before it is born, and is precociously involved in the initial siege of a representation of what its presence will be in the lives of these people, and what place it will occupy where it is received^(20)^.

There is a dialectic between the imaginary baby, the effect of the family’s projections and idealizations, and the real baby with its own capacities, and nurses must explore how the family assumes this discrepancy^(8)^. It is essential that this child is recognized in its difference from the imaginary baby, so that it can appropriate the marks of its history without simply repeating the past^(8)^.

For these reasons, the child, immersed in the symbolic register of language, makes a symptom, and this reveals the parental truth, since it is allocated to a family and constitutes a problem for each of its parents before it is born, since a destiny is outlined, in which the basis of the oedipal plot is already in place^(20)^. In other words, the child’s symptom is the condition for responding to what is symptomatic in the family structure^(19,20)^.

Therefore, there is a relationship between the place where the child is positioned by their parents and the symptom they manifest as a response to what is symptomatic in the family dynamics^(20)^. It is in the relationship with their parents that they construct their symptom, incorporating their subjectivity and freeing themselves from the mortifying siege reproduced by them^(20)^.

In this context, nurses can intervene by considering the conditions given to this child to occupy a place in language and how they respond to the demands of the Other^(19,20)^.

By way of example, behavior that is considered agitated can often be the result of personal and/or family conflicts, which the child is not yet able to express verbally. In other words, it will be in the interaction with third parties, in this case the nurse, that the child will stage the appropriation of the Other’s signifiers and their symptom should be perceived as an expression of language that informs about a parental fantasy^(20)^.

Therefore, the nurse’s job is not to modify the manifest behavior, but to work with the subject’s subjectivity, providing unique listening, understanding the symptoms and exploring how the child processes their life experiences^(6,20)^. Thus, their role is to accompany the children in building a form of existence that sustains them^(23)^.

Consequently, the nurse’s clinic based on the theoretical reading proposed here has no intention of regulating behavior, but rather of reading the children and their symptoms, rescuing the symbolic place they occupy in the family myth and allowing them to be structured as a subject^(8,20)^.

It can be seen that the symptom incorporated into the child’s body is a symbolic representative of the parents’ psychic dynamics, which can’t drain away in any other way^(20)^. Intervening with children from this perspective implies recognizing the marks of the Other on their bodies^(20)^. Therefore, nurses should also consider assisting the parents so that, as well as understanding the family myth, some effect can be produced in the family’s discourse and the possibility of a relationship of trust can be built that keeps them committed to the therapeutic work aimed at the child^(8)^.

However, there is no fixed rule for including parents in treatment. It is necessary to listen and act according to the specific nature of each case, and the nurse must assess how this entry will take place, which depends on the place the child occupies in the family myth^(8)^. It may be advisable to carry out one-off interviews with the parents, interviews with the parents at the same time as nursing consultations with the child, or even to include them in them^(8)^. For example, if the child is kept quiet by their parents, they need to be seen in their absence, so that they can express themselves subjectively in order to work through their symptoms and find a way of structuring themselves as subjects.

It is worth emphasizing that it is not a question of considering the family myth purely as a problem that could be avoided^(8)^. The nurse, as part of this care, takes the family myth as an alternative in which the children become their own and with them, giving them a singular and irreducible difference, instead of occupying, for most or all of their life, a place that they have been given, in which they make no changes^(8)^.

Therefore, the expected result of this care is not a cure, but the possibility of transforming the subject, through the formation of a radically new experience of themselves, considering all the signifiers of the family myth that they carry^(24)^.

Thus, considering prehistory is important, but care must be taken, as giving it a leading role constitutes reductionism, causing professionals to listen to and attend only to what comes from parents, grandparents and other people who permeate the family myth^(8)^. In other words, it is through the truth of the parental discourse that a demand arises, which must be seen by the nurse as a symptom presented by the parents, which never corresponds to what the child imprints under the transferential effect^(20)^.

The transferential relationship emerges as children addres their own symptom to the nurse, disconnected from the demand presented by the parents^(20)^. The professional must consider that the parents’ requests almost never corresponds to what the child needs, as their own demand will only be discovered when they talk about their symptom under the transference effect with the nurse^(20)^.

The opposite should also be considered: the reverse reductionism leads to focusing exclusively on the phantasm produced by the child, ending the treatment in their imaginary processes^(8)^. As a result, it excludes consideration of the discourses that circulate in the family about the child, who they replace, what places they inherit, etc^(8)^.

It is therefore up to nurses to work with the dialectic between considering the prehistory and, at the same time, breaking it. In order to do this, children must be listened to as their linguistic acquisitions and psychomotor manifestations develop, so that they can become a subject and not just the adult accompanying them^(5)^. It is essential that the professional observes the paths of autonomy and the search for differentiation that the child builds towards independence from the environment, which hardly happens without a certain amount of aggression directed at the family itself, such as when, during a game, the child kills his mother^(25)^.

Therefore, in their initial nursing assessment, nurses working within this theoretical framework have the task of investigating whether there is suffering on the part of the child, as a condition for treatment, and it is essential that their symptoms are different from those found in their parents^(20,26)^. This is because the child is responding to a desire that predates their existence and, therefore, it is necessary to know whether they react in a traumatic way to the attempt to respond to this place imposed on them by their parents^(20)^.

Through these strategies, children are recognized as a priority, as they are one of the most vulnerable groups in humanity^(5)^. Their dependence on adults, especially their families, requires that the perspective of comprehensive health care guarantees well-established links between the child, the family and the professional responsible^(5,27)^. By acting in this way, nurses enable the transformation of the subject, achieved by building a new experience of themselves, taking into account the family myth in which they are inserted^(24)^.

The way of care presented here can be considered by nurses in a variety of contexts, such as primary care, which is considered the gateway for cases of childhood mental disorders, and also in specialized care points of the RAPS, such as the CAPSij^(12)^. There, nurses must work in partnership with the family, taking into account the guidelines of the biopsychosocial care model, the principles of the SUS on comprehensive health care and the policies aimed at assisting children^(11,15)^.

## FINAL CONSIDERATIONS

This study responded to the objective of reflecting on the contribution of the concept of family myth to nursing care for children in psychological distress, by considering the importance of the family in child mental health care and how nurses can insert themselves into this context of action, appropriating a theoretical framework that enables them to understand the conflicts that arise in the structuring of a subject, as a possibility for producing health.

There is a consensus in current policies that the family is a key axis in childcare. Against this backdrop, it is appropriate to consider the dialectic present in the family context, which does not always behave like the harmonious unit described in current policies and literature, and is even the cause of essential conflicts for the structuring of the child as a subject. To think of the family in this way is to consider the concept of the family myth, made up of the signifiers presented to the child in their first moment of self-elaboration.

The contribution of the family myth to nursing care for children in psychic distress consists of considering the signifiers that mark their psychic structuring, the place in which they were placed by their family and the way in which they construct and elaborate a place of their own. In other words, nurses need to grasp the fragments of the family myth so that, when caring for the child, they can discover which of its aspects produce the marks that build their symptom. Based on this, care for the child is directed in such a way as to break with the place that has been assigned to them, so that they can be structured as a subject in their subjectivity.
